# Monthly Summary

**Published:** 1886-12

**Authors:** 


### Monthly Summary.

---

PIN-HOLE CAVITIES AND "CARIES INTERNA."—In practice we not uncommonly meet with cases where a very small opening through the enamel is the only external indication of a very large cavity in the dentine, and even when the enamel is apparently not yet broken through, we may find, on cutting into it, a cavity already forming, or at least a considerable softening in the dentine, giving rise to what I think has often been mistaken for "caries interna." We frequently ask ourselves the question: Can the small particles of food which may enter through so minute an opening bring about so extensive a decalcification? Recent observations have rather inclined me to the view that it must, or at least may, be so.

In explanation of the first case, I have seen the same appearance exactly in pulpless teeth, and have produced it artificially. I have a second temporary molar which was exposed for seven months in a fermenting solution. It shows three pin-holes on the grinding surface, one of them extending nearly to the pulp and undermining the enamel in all directions. Here there was nothing present to produce the effect described, excepting the chemico-parasitic factors.

In explanation of the second case, I have observed that acids may work their way through enamel in sufficient quantity to produce a softening of the underlying dentine, without completely breaking down the enamel, or revealing a cavity to the naked eye or a blunt instrument.

I have specimens in which the dentine is infiltrated with micro-organisms and beginning to dissolve, while the enamel covering is still not completely disintegrated. In all cases of this nature inter-globular spaces naturally play a very great role. In teeth of very inferior structures the dentine is in some places a mere framework, which is quickly softened, even by very weak acids. It must, furthermore, be borne in mind that in mastication food is subjected to considerable pressure, by which it may be forced through the smallest cracks and openings. We have all seen how particles of food may find their way to the very apex of a root canal which we have under treatment, if we allow it to remain open for a day.--*Independent Practitioner*.

---

PREVENTIVE DENTISTRY.—That we, as a profession, make our living from the results of the negligence of our patrons in not properly caring for their mouths, all must admit. Of a thousand carious cavities filled, nine hundred and ninety-nine might have been prevented from decay; and of the thousands of fillings made, how many of them prevent the loss of the pulp, or the teeth themselves, and really prevent the use of crockery substitutes? Our artificial way of living is largely responsible for these ravages by decay. The chemically prepared groceries, and the fermented fruits and vegetables, transported from long distances and different climates, added to soft, sloppy cooked mixtures of lemons, eggs, milk and sugar, enter now largely into our daily diet; the tendency being all the time towards preparing dishes that will melt in the mouth, rather than those that are healthful to the dental organs, and thereby healthful to the body, such as tough meat, hard bread, and simply prepared articles that require physical force in mastication. The former articles are not insalivated as they pass through the oral cavity; and the secretions that are furnished by the glands and membranes, from all such diet, are really of a chemical nature, which dissolve, not only any food that may be left in the mouth or between the teeth, but the teeth themselves. This food not having any physical resistance, mastication is not necessary, and insalivation not important, and the secretions of the organs of the economy are acrid; added to these unhealthy surroundings, the period of rest or stagnation, if I may so call the interval between meals and during the night, these impure secretions become more erosive by being retained in the warm surroundings, and these mephitic odors and gases, are very perceptible to our associates, although the patients may pride themselves upon being very tidy and cleanly about their mouths. It is only necessary, to convince to the contrary, one who imagines that his mouth is in good hygienic condition, to let him pass a thread or quill tooth-pick between the teeth in unfrequented regions, and to put it to his nose, when he will understand why decay could so readily occur. It is not the perversity of human nature, but the ignorance of the importance of thorough cleanliness, that makes so much work for dentists. A proper, thorough and skillful application of the tooth-brush is practiced by the fewest number. It is really a work of art to cleanse teeth, and the oral cavities, tonsils, fauces, so they will bear a critical examination. The Chinese have a professional, whose duty it is to pick and cleanse the teeth, while caring for the other special organs of the head.

The natives of India in performing the mysteries of their toilet, while brushing the teeth and mouth, run the primitive banyan-root brush down the throat so far and make such retching, hawking and spitting, that the uninitiated would imagine that some terrible calamity had befallen the victim's gastric region. Instruct your patients to brush after meals and before going to bed, with water; and often hot water is required. Use a medium sized brush of medium strength of bristle, with an upward and downward motion from the gums to the edges of the teeth, making the bristles act as so many tooth-picks, forcing and lifting out particles of food. Do not recommend the frequent use of any dentifrice, for to their excessive use, can be traced nearly all cases of abrasion. Our whole duty is to do our operations the best that circumstances and attending conditions will admit, and then give the patient careful instruction to prevent all further destruction of these most valuable organs; for, if like conditions are allowed to continue, similar destruction will be inevitable. Dentistry, like medicine, should seek to prevent these large mouths full of dentistry, and should prevent decay in the deciduous teeth.

The dentist should instruct the patient in the care of the teeth, and step by step, as the new ones come forward, they should be cleansed, brushed and picked, and cared for, in such a manner that decay would not occur. Then in the ideal future, when we can see our patients with a sound mind, in a sound physique, supported by food masticated with sound teeth, the pinnacle or highest result of dentistry will have been reached.—W. N. MORRISON, *in Archives of Dentistry.*

---

POWER OF AN OPERATOR OVER A PATIENT UNDER THE INFLUENCE OF NITROUS OXIDE.—I have been using nitrous oxide gas in my practice for a number of years. For the first two or three years I had but indifferent success, especially when called upon to remove the wisdom-teeth or second molars. To insure the mouth being open, I used to employ a rubber gag, sometimes substituting for it a large cork with a string attached. In a majority of cases, when the gag was taken out the jaws closed somewhat and remained rigid; no proper amount of force sufficing to open the mouth until the effect of the gas was nearly spent. I finally declined to administer gas for extracting teeth back of the first molars.

Requiring the extraction of a tooth from my own mouth, I went to a brother dentist and had the gas administered. While taking it and while under its effects I was perfectly conscious; heard every word said, but was powerless to move a muscle. I knew when the inhaler was removed and the forceps taken up and adjusted, but felt no pain. The tooth was broken off, and two more ineffectual efforts were made to extract it. Still there was no pain. At this instant I regained control of myself, retaining a knowledge of all that had transpired.

This experience impressed me with the conviction that if patients heard and understood they could be made to obey. It had been a habit with me to test the condition of my patients during the inhalation of the gas by requesting them to raise the left arm. They usually responded, some continuing to raise and lower the arm until the effect of the gas passed off, seeming to be impressed with the idea that they must do so.

The next occasion I had to administer gas I made the usual examination of the mouth, and, after learning which teeth I was required to remove, I turned abruptly to the patient, and said, "If, while you are under the influence of the gas, you hear me request you to open your mouth, do so. You can if you understand now that it will be necessary." I said nothing about raising his arm, but just as I thought he had inhaled enough gas I said, "Raise your left arm," and was surprised to see that, instead of doing so, he promptly opened his mouth wide. As his breathing indicated that he had enough gas, I dropped the inhaler and extracted the teeth. After he regained consciousness I said, "Did you hear me ask you to raise your arm?" He said, "I heard you tell me something, and as you had charged me to open my mouth if asked to, I thought that was what you wanted."

I have taken advantage of this experience ever since—some three years—and have yet to meet the first failure. After charging a patient to respond by opening the mouth when told to, I proceed to administer the gas, and just before he passes under its influence I say, "Remember and open your mouth when I tell you to." Thus the last thing the patient thinks of is opening his mouth, and almost invariably it is the first thing he does. I have tried the command without previous instruction, and failed to obtain a response. I think this suggestion worthy of investigation by those who use gas.—*W. C. Bunker, Oregon, Ill.*

---

HYDRONAPHTHOL—A NEW ANTISPETIC.*—Hydronaphthol is a non-poisonous, non-corrosive antiseptic and disinfectant. It has a slightly perceptible aromatic taste and odor,

* I am indebted to Dr. George R. Fowler, of Brooklyn, for the facts regarding the composition, properties and relative strength of hydronaphthol in comparison with other antiseptics.

and crystallizes in scale-like clino-rhomboid lamine, of a silvery-white or grayish hue, resembling zinc-bronze. It is a derivative of the hydroxyl substitution of naphthalin. Within the last two years it has been discovered that this substance possesses antiseptic properties, and the claim is made that it is from ten to fifteen times more efficient than carbolic acid, being the most promising antiseptic of the phenol series. As an antiseptic, it is about one-fifth as powerful as the mercuric chloride; from one and a half times to double the strength of iodine, and four times as strong as sulphurous acid. Carbolic and salicylic acids follow in the list of antiseptics, and it is thirty times as powerful as the latter.

Hydronaphthol possesses many advantages over carbolic acid, for besides its being non-poisonous and non-corrosive, it is a non-irritant. Its odor is not sufficiently strong to disguise that of putrefaction, as it happens with carbolic acid. It is not decomposed or rendered inert by the products of putrefactive decomposition, and this property renders it invaluable in disinfecting pulpless teeth, besides its being far more stable than carbolic acid, not being volatile at ordinary temperatures.

Hydronaphthol is only soluble in water to the extent of one part in one thousand, but even in this proportion it is a powerful antiseptic, while a solution of one to ten thousand prevents putrefactive decomposition. It is freely soluble in alcohol, ether, benzol, glycerin and the fixed oils.

I have been experimenting with this substance, and have kept a careful record of the treatment of twenty-three cases of pulpless teeth, and the satisfactory results so far obtained prompt me to call the attention of the profession; through the *Dental Cosmos*, to the valuable characteristics of this new antiseptic.

My modus operandi is as follows: Dissolve the hydronaphthol in glycerin, and introduce a saturated cotton pellet into the pulp-canal, which has already been prepared in the usual way. One application alone seems to act as a radical specific. All soreness, if there be any, disappears in the course of twenty-four hours, as also all traces of putrefaction. At the second sitting, remove the dressing, syringe with tepid water, and proceed to fill the canal permanently with cotton, saturated with oxyphosphate and hydronaphthol. This is accomplished by mixing some thin oxyphosphate, to which is added one-fourth the quantity, by bulk, of hydronaphthol.

In cases of chronic alveolar abscess, force the hydronaphtholated glycerin through the point of discharge, using for this purpose Dr. R. W. Starr's exhaust syringe, which is very essential in the proper treatment of alveolar abscesses, and fill the canal as before stated. I have been successful in curing alveolar abscesses of long standing in this manner, and have not had a single case of recurrence.—*Francis Eschauzier, D. D. S., Brooklyn, N. Y.*

---

A CURIOUS CHRISTMAS BOX.—The relator of the following amusing incident was once visited by a former colleague, who asked how he could give his wife a set of artifical teeth on Christmas Eve without her knowing he was going to do so until the presentation.

The friend assured him that if his wife had but one hollow tooth, he could easily obtain an impression without her knowledge.

Accordingly the lady, who was suffering from toothache was brought by her husband to consult his dentist friend. After examination, the latter told the lady he could relieve her from any further pain, but in order to get the stopping properly prepared, he would be obliged to make a wax impression of the tooth he was to stop.

The lady was alarmed. and thought this would be a very painful operation, but consented to submit when her self-sacrificing husband promised to be experimented on first by way of re-assuring her.

The following day the lady again appeared, alone this time, and requested the dentist to make artificial teeth for her husband, to replace those he had lost. She also wished to know if he could do so without her husband's knowledge, as she particularly wished to give them to him as a present on Christmas Eve.

Just before the festival, the two packets containing the teeth were sent off, that with the lady's teeth was taken through the post to the business office of the husband, while the other was addressed to the lady, at their private residence.

The evening came, and with it the double surprise.

Some days after the husband visited his friend and assured him that he should never as long as he lived forget the amusing and astonishing exchange of presents which had taken place on Christmas Eve.—*Zahntechnische Reform—British Journal of Dental Science.*

